# Access to care for childhood cancers in India: perspectives of health care providers and the implications for universal health coverage

**DOI:** 10.1186/s12889-020-09758-3

**Published:** 2020-11-03

**Authors:** Neha Faruqui, Sarah Bernays, Alexandra Martiniuk, Seye Abimbola, Ramandeep Arora, Jennifer Lowe, Avram Denburg, Rohina Joshi

**Affiliations:** 1 Sydney School of Public Health, The University of Sydney, Sydney, NSW Australia; 2 London School of Hygiene and Tropical Medicine, London, UK; 3 George Institute for Global Health, Sydney, NSW Australia; 4 Dalla Lana School of Public Health, University of Toronto, Toronto, Canada; 5 Cankids … Kidscan, New Delhi, India; 6 Max Super Speciality Hospital, New Delhi, India; 7 Division of Haematology/Oncology, Hospital for Sick Children, Toronto, Canada; 8 Faculty of Medicine, University of Toronto, Toronto, Canada; 9 Faculty of Medicine, University of New South Wales, Sydney, Australia; 10 George Institute for Global Health, New Delhi, India

**Keywords:** Qualitative study, India, Childhood cancer, Health care provider, Accessing care, Barriers, Universal health coverage

## Abstract

**Background:**

There are multiple barriers impeding access to childhood cancer care in the Indian health system. Understanding what the barriers are, how various stakeholders perceive these barriers and what influences their perceptions are essential in improving access to care, thereby contributing towards achieving Universal Health Coverage (UHC). This study aims to explore the challenges for accessing childhood cancer care through health care provider perspectives in India.

**Methods:**

This study was conducted in 7 tertiary cancer hospitals (3 public, 3 private and 1 charitable trust hospital) across Delhi and Hyderabad. We recruited 27 healthcare providers involved in childhood cancer care. Semi-structured interviews were audio recorded after obtaining informed consent. A thematic and inductive approach to content analysis was conducted and organised using NVivo 11 software.

**Results:**

Participants described a constellation of interconnected barriers to accessing care such as insufficient infrastructure and supportive care, patient knowledge and awareness, sociocultural beliefs, and weak referral pathways. However, these barriers were reflected upon differently based on participant perception through three key influences: 1) the type of hospital setting: public hospitals constituted more barriers such as patient navigation issues and inadequate health workforce, whereas charitable trust and private hospitals were better equipped to provide services. 2) the participant’s cadre: the nature of the participant’s role meant a different degree of exposure to the challenges families faced, where for example, social workers provided more in-depth accounts of barriers from their day-to-day interactions with families, compared to oncologists. 3) individual perceptions within cadres: regardless of the hospital setting or cadre, participants expressed individual varied opinions of barriers such as acceptance of delay and recognition of stakeholder accountabilities, where governance was a major issue. These influences alluded to not only tangible and structural barriers but also intangible barriers which are part of service provision and stakeholder relationships.

**Conclusion:**

Although participants acknowledged that accessing childhood cancer care in India is limited by several barriers, perceptions of these barriers varied. Our findings illustrate that health care provider perceptions are shaped by their experiences, interests and standpoints, which are useful towards informing policy for childhood cancers within UHC.

**Supplementary Information:**

The online version contains supplementary material available at 10.1186/s12889-020-09758-3.

## Background

Nearly 90% of children with cancer live in low- and middle-income countries (LMICs), where weak health systems contribute to lower survival rates relative to high-income countries [1]. India is an example of a LMIC setting where in 2017, childhood cancer was the 5th leading cause of death amongst 5–14 year olds [2]. The Indian health system is fighting an increasing rate of non-communicable disease (NCD), but with less than 1.3% national expenditure of gross domestic product on public health care, there is a resulting poor quality of service provision [3]. In addition, there is a growing dominance of the private health care sector in India, requiring out-of-pocket expenditure for the majority of the population. Regrettably, private health services are mostly unattainable in the rural areas, leaving 70% of the population unable to access such health services [3]. Childhood cancer care is only available at the tertiary level and since most functioning cancer centres are located in metropolitan cities, the rural sector remains deprived of this service [4]. This contributes to a delay in presentation where for example, it was found that 80% of children with neuroblastoma [5] and 40–60% of children with Hodgkin lymphoma [6, 7] presented in advanced stages at treating hospitals.

While there is a national policy for cancer prevention and control, India does not have a policy framework specifically addressing childhood cancer care. However, the country has supported the notion of ‘health for all’ since independence and has increased commitments to improve the public health system, which is the backbone for Universal Health Coverage (UHC) [8]. UHC is defined as individuals and communities having access to necessary health services (prevention, treatment, rehabilitation and palliative care) without suffering financial hardship [9]. Efforts to achieve UHC have largely focused on “financial hardship” reforms [10–15]. This includes India’s efforts to launch Ayushman Bharat in 2018; a central government health protection scheme, which includes some financial coverage for childhood cancer treatment [16]. While the scheme addresses barriers to affordable health care, the supply side of health services still needs to be strengthened – i.e. health services need to be available with equitable facility distribution, service quality, and appropriately trained staff to ensure that families are treated with dignity and respect [17]. On the demand side, families need to be able to physically access health care and have appropriate knowledge and awareness (health literacy and beliefs) in order to seek and adhere to care [17].

Health reforms often address the tangible elements of a health system (e.g. service delivery, information, or health workforce). But reforms also need to focus on intangible components such as the interests, ideas, power and organisational culture (e.g. social constructs and behaviour) influencing the system [18]. Qualitative methods can help generate evidence on such tangible and intangible components, by exploring the perspectives of stakeholders such as health care providers on barriers to accessing care [19–21] and to achieving UHC [18, 22–27], which can influence decision-making and policy dialogue [18, 28]. However, such evidence is scarce in the literature on barriers to accessing care for childhood cancers in LMICs [29–32] and of the few qualitative studies in India regarding cancer care [33, 34], none focused on paediatric oncology and the health care provider’s perspective. This study aimed to gain the perspectives of health care providers on challenges to accessing care for diagnosing and treating childhood cancers in India, and the implications of these perspectives for efforts to achieve UHC; an important means to adequately ensure optimal health care for children with cancer.

A similar qualitative study on barriers to accessing childhood cancer care from the perspective of caregivers was conducted by the lead study investigator and team [35]. The results indicated that time to definitive diagnosis and treatment, social supportive care and ongoing emotional and financial impacts were the major themes affecting access to cancer care. The current study investigates the complementary perspectives of health care providers regarding barriers to accessing care for childhood cancers.

## Methods

### Sampling and recruitment

This study was conducted in New Delhi (North India) and Hyderabad (South India) as the lead study investigator (NF) and interviewers were comfortable with the languages spoken in these cities (Hindi, Telugu and Urdu). Although both are large urban metropolitan cities, they are different as Delhi caters to patients from loco-regional as well as distant areas and Hyderabad caters mostly to patients from loco-regional areas.

Seven tertiary hospitals treating all childhood cancers across the two cities, were purposively selected to provide an adequate representation of the public sector (*n* = 3), private sector (*n* = 3) and charitable trust sector (*n* = 1). Public hospitals provide free or subsidised services, private hospitals provide paid services or subsidised services for a small percentage of patients who are below poverty line and charitable trust hospitals, although not government owned, provide free or subsidised services through external donor funding.

The inclusion criterion incorporated health care providers involved in the management of children with cancers at the participating treating hospital, who could speak a local language known to interviewers (Hindi, English, Urdu, Telugu). We aimed to recruit different levels of health care providers through stratified purposive sampling, reflecting the range of roles involved in paediatric oncology care within the tertiary health system. The local head paediatric oncologist of the hospital unit referred the interviewers to relevant health care providers in the hospital to invite them to participate in the study. Although we had recruited through stratified purposive sampling, due to the heavy workload in hospitals, participants were often recruited based on availability and hence we had to adopt a technique of convenience sampling as well.

### Data collection

All interviews were conducted by female interviewers (NF and a research assistant) fluent in the local language and trained in qualitative research. Researchers did not have an established relationship with the participants prior to the data collection. Participants were interviewed using a semi-structured interview guide designed by the authors, based on the chronological order of a typical patient’s journey i.e. from symptom onset to reaching care, getting diagnosed, treatment initiation, treatment continuity and palliation (Additional file 1, Table 1). Questions were asked regarding the health care provider’s opinion of the individual and health system factors affecting access to care along the journey. Follow up or probe questions were asked as necessary to gather pertinent detail and were used flexibly depending on the answer to the open-ended nature of the questions. Although interview questions predominantly focused on the challenges, we also fostered the opportunity for participants to narrate experiences and opinions of facilitators or solutions to the challenges. Field notes were taken and data was collected to theoretical saturation, where no new information was observed. All interviews took place in a private setting within the hospital.

### Data analysis

Participants and hospital names were de-identified prior to analysis. All participant interviews were transcribed verbatim and translated into English by a qualified transcriber. There were no further comments or corrections required from participants. A set of deductive codes and sub-codes pertaining to barriers in accessing care was first developed by NF and JL using Donabedian (1988) and Penchansky’s (1981) framework, as these frameworks broadly depict barriers to accessing healthcare in general [36, 37] (Additional file 2, Table 2). An iterative and inductive approach to content analysis was then undertaken, where new codes were identified within the data. Categories relating to facilitators or solutions were also identified and coded in. An interpretivist position was applied to the analysis focusing on the participants’ perspectives so that emerging categories and themes were conceptually understood in relation to participants’ experience, role and positioning of their opinions. NF, SB and SA conducted a triangulated analysis to develop concept categories and themes. NVivo 11 software was used to manage and code the data.

## Results

**Fig. 1:**
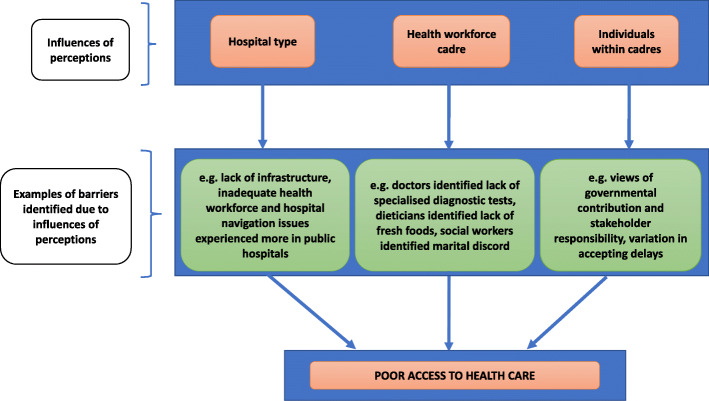
Conceptual illustration of influences of perceptions and examples of barriers identified which lead to poor access to health care

Twenty-seven health care providers treating childhood cancers or involved in the care of childhood cancers were interviewed which included eight oncologists, five nurses, five social workers, four senior resident doctors, two dieticians, two psychologists and one radiotherapist. Interview duration ranged from 10 to 60 min, where majority of the interviews lasted between 25 to 45 min. Barriers to accessing care were related to hospital service provision, personal factors of families seeking care and factors external to the health system. However, as shown in Fig. 1, barriers were reflected upon differently based on the following influences of participant perception: 1) hospital type – public versus private or charitable trust, 2) health workforce cadre and nature of engagement with patients, and 3) individual opinions/perceptions within cadres. The results of this study are thus framed and presented through these three key influences.

To situate the perspectives of participants, we first provide an overview of the barriers that participants identified and responded to: Participants mainly reported barriers at the treating centre such as lack of infrastructure, inadequate health workforce, poor social supportive care, inappropriate food and lodging and financial problems (barriers are covered in more detail in subsequent results sections). They however also acknowledged several barriers from symptom onset until families reached the treating centre. This included geographic challenges, paucity of diagnostic facilities, poor sensitization of paediatricians, financial problems, poor caregiver literacy, sociocultural beliefs and inappropriate healthcare-seeking practices. Some of these demand-side caregiver barriers such as literacy, financial problems and sociocultural beliefs were still prevalent upon commencing treatment.

Participants’ perspectives were similar across both cities and are therefore presented collectively in the results. However, barriers were experienced more in public hospitals than private/charitable trust, and thus in addition, more participants were recruited from public hospitals (*n* = 17) than private (*n* = 7) and charitable trust hospitals (*n* = 3).

### Influence of hospital type

The type of hospital in which a health care provider worked, influenced their perceptions of barriers. The charitable trust hospital and private hospitals generally had better health facility infrastructure and organisation. Health care providers indicated that this was instrumental in providing definitive and timely diagnosis and treatment. Health care providers in public hospitals highlighted the barrier of insufficient infrastructure in catering to the large patient population, such as the number of beds, rooms for examination or consultation, diagnostic equipment and inadequate health workforce; all of which resulted in long waiting times and delay to definitive diagnosis and treatment. Health care providers in the public hospitals also indicated the need for more nurses (especially trained in oncology) to help with the patient load. This was contrasted by perceptions on better health workforce training and job satisfaction expressed by nurses in private hospitals:

*We are on the right path and right hands due to the training and education. Everything is in a proper way. And we feel that yes, we are in advanced hands (…) After the treatment is completed, [families] come and say sister whatever you told us, everything has happened. So then we feel proud. (Nurse 1N, private hospital 1)*

For health care providers in the charitable trust hospital and private hospitals, the focus was on challenges for the family in adjusting to treatment procedures such as chemotherapy and concerns over side-effects and frequent testing. Whereas, participants in public hospitals highlighted barriers which were focused on the health system such as the additional efforts required for patients to navigate their way through the hospital, finding the right testing and treatment rooms, working through the hospital’s administrative bureaucracy and finding accommodation for the duration of the chemotherapy cycle. This meant that public hospital health care providers had less time to develop a good rapport with families and address queries about specific treatment issues. Yet, health care providers in public hospitals highlighted the importance of service delivery with good communication skills and compassion, particularly for those families who already undertook a demanding journey during the referral pathway:

*It is very important that the [treating team] should be compassionate. If that is not there, there is a huge amount of loss to follow up which is not good particularly in cancer care. (Oncologist 1O, public hospital 2)*

*There are some doctors who speak very badly. So they feel that it would be better not to get the treatment done from here and instead go somewhere else. If a patient asks a question they say “don’t ask anything now, we will talk later, I have already written on your OPD card. Don’t you understand if you are told once?” This is how they speak. This is not right. (Social worker 1S, public hospital 3)*

Public hospital health care providers in particular, highlighted inadequate social supportive care as a barrier in engaging with families and delivering effective comprehensive care. This included follow up, guidance for accommodation, nutrition counselling and general assistance in patient navigation. Although participants in all hospitals felt training in psycho-oncology care and awareness was important, most (especially public hospitals) did not have facilities or an adequately trained workforce to deliver social supportive care. Social workers, counsellors from non-governmental organisations (NGOs) or private student researchers conducting studies in psychology were instead sometimes recruited to provide counselling. Participants acknowledged the importance of mental health care, *“stigma associated with psychiatric care” (Psychologist 1P, public hospital 3)* and the necessary awareness for families:

*Families do not know that anything like mental health care exists. They do not know about the existence of psychology. They do not know that there is something like caregiver burden. So there is a complete lack of awareness. (Psychologist 1P, public hospital 3)*

In addition, palliative care was a neglected area across all hospitals, except one public hospital which had a special integrated paediatric palliative care program. The other hospitals lacked adequate services due to insufficient facilities, patient overload, time constraints and scarce expertise meaning inadequate emotional and clinical support from curative to palliative care transition.

In public hospitals, the intense patient navigation problems, time-consuming and laborious tasks and the solitary nature of the treatment experience meant that families usually required more than one caregiver present at the hospital or at least an active male member. Families, especially those facing barriers due to low socioeconomic backgrounds and limited education, were often left *“perplexed and confused” (Psychologist 1P, public hospital 3)* during the diagnosis and treatment experience. The family’s breadwinner often expended time and energy, leading to indirect costs such as emotional exhaustion; setting a negative state of mind through cyclical episodes of worry and anxiety. However, as private hospitals were relatively well equipped, health care providers could deliver more support and empowerment to families with one caregiver, meaning the breadwinner of the family could return to work; reducing indirect costs.

### Influence of cadre and nature of engagement with patients

The nature of engagement with patients and their families, often determined by the cadre of each health care provider, influenced their knowledge and perceptions of barriers. This in turn framed participants’ priorities of what was important to families and to service delivery. For example, clinicians (oncologists, senior residents and nurses) provided an overall insight into the journey of the family’s diagnostic and treatment experience such as financial barriers, lack of specialised diagnostic investigations such as genetic testing, ability to identify a few sociocultural factors, limited human resources in hospitals and the need for child friendly services. Dieticians noted some common socioeconomic barriers but notably mentioned the lack of cooking facilities for families with inappropriate accommodation, and the need for a fresh and healthy diet for sick children:

*If the child is really malnourished, and they cannot afford green leafy vegetables, we actually ask them to go for the seasonal thing. So, in winters the green leafy vegetables are way cheaper than other vegetables. (Dietician 1D, public hospital 3)*

Psychologists equally had a deeper insight into barriers, determined by their role, such as barriers affecting the mental well-being and treatment compliance. Social workers however were able to maintain an engaging role on a day-to-day basis for the treatment duration. Their proximity to non-clinical aspects allowed them to gain a unique and in-depth insight of barriers that were a product of the intersection between the social, relational and economic concerns of families.

*Families often tell me “We don’t know what to do. Our mind is not working. We don’t have money, we don’t have proper sleep since one month. And we don’t even have proper [clothes] to wear”. Some are in a miserable condition. (Social worker 1S, public hospital 4)*

Social workers could capture distinct detail and nuances of barriers such as socio-cultural reasons underpinning discontinuation of treatment between male and female patients, which was unusual across the other cadres. A reason for abandonment of treatment of the girl child was sometimes due to social factors, such as refusing an amputation so that marriage prospects were still tenable. Another barrier noted by all social workers and two clinicians was marital discord due to the various pressures families faced. This was sometimes a result of differences in opinion between caregivers regarding how to prioritise and use their remaining scarce resources, including meeting the care needs of their other children. Marital discord could also arise due to the mental health impact of the child’s medical situation on the other caregiver or spouse. This was more likely to affect mothers. In cases where relationships broke down, the woman encountered further economic and social pressures as they had to find alternate sources of income. Additional problems included finding safe and secure accommodation at night.

Such barriers, resulting from social and relational pressures, were seldom explained in detail by providers of other cadres. Some social workers also described their open dissatisfaction with certain clinicians in the delivery of care, citing incidents they had witnessed, which they considered to negatively impact family compliance and confidence. While this cadre provided valuable insights, they also felt that their role was somewhat under-recognised in providing care:

*Sometimes some people don’t know what a social worker is or what counselling is. Problems are there, productivity is there, but organisations don’t know how to utilise social workers in the correct way. (Social worker 1S, charitable trust hospital 5).*

Although these insights were not only provided by social workers, they were more commonly prioritised by this group. A few participants from other cadres acknowledged the important need of social workers in providing care, which could mitigate the social consequences disrupting the families’ engagement in care. This was acknowledged as a valuable contribution, which reduced the workload burden for clinicians:

*I would like good support from social workers (…) Because now me and my junior doctor are the coordinators. We are struggling with raising the funds and everything. So [we need] someone like a social worker who can look into these things, so that we can concentrate more on medical things and research (Oncologist 1O, private hospital 6)*

### Influence of individual perception within cadres

While perceptions varied by hospital type and cadre, they also varied within cadres and roles based on their individual perceptions and expectations that framed how they viewed the system. An individual’s perception and expectation of what should determine an “acceptable” delay, who is responsible for addressing barriers and what should the government’s role and priorities be in relation to childhood cancer, influenced the participant’s reasons for delay and quality of service delivery. Firstly, on what determines “acceptable” delay, some participants in private hospitals were satisfied with the diagnosis and treatment progress upon families reaching the hospital, and a few participants in public hospitals emphasised there is not much delay once families reach the right department. One participant stated that long waiting times are not necessarily a delay barrier and depending on the cancer type, this waiting time is acceptable for *“some patients who can afford to be delayed”(Oncologist 1O, public hospital 3)*:

*There are tumours which can wait easily for 2, 3, or 4 weeks and nothing will change. We don’t really rush them for an early diagnosis. It makes no sense to overload a system to rush a diagnosis which is not urgent. (Oncologist 1O, public hospital 3)*

Whereas most participants, such as a senior resident (below), stated that delay indeed occurs in public hospitals and did not acknowledge any cancers for which delay was “acceptable”:

*Getting dates for biopsies, getting dates for medical tests will take waiting time, around 1 month or 45 days of waiting time. I think in that period certain cancers that are localised and operable, get advanced by then. (Senior resident 1R, public hospital 3)*

Secondly, on responsibility for addressing barriers: this was framed in terms of the relative role and expectations of various stakeholders, where a few participants felt clinicians played a vital role:

*Doctors are the ones who educate the patients (…) [Doctor] Ma’am mainly helps the patients who cannot afford the treatment (…) So for those patients [Doctor] Ma’am helps to fill out the forms for the Prime Minister Relief Fund and gets it deposited. (Nurse 2N, private hospital 1)*

Other participants felt the responsibility resided with the government, communities, NGOs or companies engaging in corporate social responsibility – all of whom in different settings and at different times provide patients with subsidised diagnostic and treatment services, free medicines, food, accommodation, provision of counselling and help with navigating the system. While many acknowledged the significant role of NGOs in supporting access to and delivery of care, some argued that NGOs should complement the system rather than create dependency, which may lead to the government abdicating its responsibilities.

Solutions to facilitating better access for families were mostly focused on improving the external health system i.e. the referral pathway and governance of the health system, which comprised of various stakeholders being responsible such as state governments, civil societies, local community groups and organisations like the Indian Medical Association. Participants’ solutions included: strengthening regional cancer centres, increase disease knowledge and awareness in the primary care setting and within communities, reforming the medical curriculum to include knowledge of childhood cancers, advancing research and training, resource mobilization for essential requirements like blood donation and recruiting cancer care coordinators at the community level to help with referrals and patient navigation.

Thirdly, within the stakeholders identified to be responsible, the government’s role and responsibility in particular was further expressed by all participants based on their individual standpoints and interests. This was especially the case for some oncologists who were more politically engaged and were assertive in their opinions of the government, expecting a stronger push for goals like UHC. This included acknowledging that insurance schemes as part of UHC can reduce out of pocket expenditure but remains inconsistent and variable throughout Indian states, leading families to even abandon treatment. Moreover, participants also recognised that strengthening primary health care remains an unfulfilled responsibility of the government, which is a crucial target for UHC.

However, a few participants recognised that the government had come a long way in improving the services, especially in the last decade with investment in basic infrastructure, the blood bank and drug provision; benefiting childhood cancer care. The participants who expressed support for the government also affirmed that free health care for children is being provided in most states. Hence *“there is a kind of unwritten universal health coverage, [although] the elements are not in place” (Oncologist 1O, private hospital 1)*. However, most participants especially across all cadres expressed dissatisfaction with the government’s contribution to improving care, attributing failure to corruption, poor public health expenditure, and competing priorities. Others also highlighted that the responsibility of improving access partly lies on the demand side i.e. with families:

*I shouldn’t feel that we can blame daily that the government is not doing this or that. It is partly the responsibility of the population as well. Their health seeking attitude I feel is not up to the mark. You can make a primary health centre, you can make a dispensary in every village but you can’t bring them out of their homes. (Senior resident 1R, public hospital 2)*

Lastly, on what should be the government’s priority moving forward: some participants emphasised that the government should be more cognisant of preventing barriers to accessing care for childhood cancers:

*There is a lot of knowledge gap in government’s understanding of cancer (…) there is a big bureaucratic red tape which actually prohibits them to talk to us. There is a lot of apathy among the [government] administration and staff, they don’t really understand that the child or parent of any cancer patient coming so far to be treated actually has a much bigger problem than you have. (Oncologist 1O, public hospital 4)*

Whereas a few other participants explained that addressing childhood cancer care could be a priority for them due to their personal interests, although it does not necessarily need to be a government policy priority:

*Why should we only be harping this for a child with cancer? I think that should be the last of the government’s priorities. Just because I am an oncologist, this is my passion. If I were the government, this has 60% chance of cure and meningitis has 90% where would I put my money? (Oncologist 1O, private hospital 1)*

## Discussion

The results of this study show that while participants acknowledged that accessing childhood cancer care in India is limited by several identified barriers, perceptions varied on the meaning of ‘accessing care’ and the associated barriers. Barriers identified by health care providers included supply-side barriers (e.g. lack of infrastructure, administrative and organisational issues, inadequate social support, lack of appropriate palliative care facilities and inadequate contributions by the government to support health service delivery) and demand-side barriers (e.g. relational and social beliefs of families, limited knowledge and awareness of the disease and health system and financial problems). These barriers were similar to those identified in previous literature [33, 34, 38–41], including the qualitative study on caregiver perspectives [35]. While this is important, the studies did not illuminate how these barriers are perceived and what influences these perceptions. This study therefore offers further nuance in understanding the barriers comprising of tangible and intangible elements. Indeed, as Gilson et al. (2012) reported, in order to support moves towards UHC, a stakeholder analysis through qualitative interviews should be conducted to understand how the stakeholder position, relative power and interests influences the perceptions and ultimately, the decisions related to policy [18].

More barriers such as weaknesses in service provision were identified within public hospitals compared to private and charitable trust hospitals, implying a worse experience for families and a greater need for reform. Perhaps for any hospital, overreliance on higher authorities to address specific barriers may limit the potential for solutions that focus on intangible elements such as organizational culture or patient-provider rapport [42]. This top-down approach may impact quality of care (due to the potential disconnect between levels of the health system), which is a major objective of UHC [43]. For example, a greater need for social supportive care for families as well as palliative care (where most of the large cancer centres lack such services [44]) was an issue acknowledged by all participants. This is critical otherwise one risks loss to follow up, abandonment of treatment and a lack of access to appropriate medication for end-of-life symptoms [38]. While top-down structural support like mental health care services and pain clinics may be provided, it also requires a bottom-up understanding of the intangible social constructs affecting individuals, such as the attitudes or behaviours shaping barriers like mental health stigma.

In addition, childhood cancer care in hospitals, like other diseases, has traditionally been led by the clinicians/physicians. Consequently, their opinion outweighs and often supersedes opinions of others in a multi-disciplinary team. Our findings show that there is often a marked incongruence in the opinions of health professionals, sometimes determined by their position on barriers to accessing care. Social workers provided different perspectives which should be valued and considered. They demonstrated ‘informal power’ through their unique experience and knowledge which has potential to contribute towards better understanding of health inequities and social determinants of health in India [45, 46]; key components of addressing UHC [47, 48]. Social workers also perhaps took the interview opportunity to express sensitive accounts of family experiences associated with their superiors (clinicians), which they otherwise probably could not openly express, given the power dynamics in a hierarchical social and health system structure [49]. Indeed, these intangible nuances are important and as Frenz et al. (2010) reported, to ‘complete the picture on UHC and equity of access’ one should understand the interactions between providers and families given that influencing factors are likely to vary [27].

Participants’ individual varied perceptions of government’s efforts, as explained by Gore and Parker (2019), may be reflective of an understated power dynamic that frontline service delivery can risk being made a political issue, such that political positioning could shape how problems are perceived [50]. Notably, participants who had opinions on government efforts were mostly oncologists, perhaps due to their involvement in high-level management and decision-making, compared to other health worker cadres who are less likely to be involved in system level change, administrative decisions, advocacy or policy dialogues. However, even within this cadre of oncologists there were variations in perception regardless of the hospital type or cadre, suggesting that an individual’s standpoints or interests may shape their views. Our findings suggest that while there is individual variation, there are sufficient patterns owing to this variation around specific issues i.e. perceptions around acceptable delays, solutions to addressing barriers and the government’s role and priorities. It is thus important to facilitate greater inclusion of various stakeholders when informing broader reforms, as has been previously reflected in LMICs [18, 51–53]. This inclusion allows for deriving commonalities as well as differences, which are useful in understanding “how to manage a reform process within a particular context” [18].

Improvements and outcomes of care for childhood cancers have resulted, in part, from involvement of and collaboration between various stakeholder groups such as doctors, parents of children who died from cancer and grassroots local foundations, who exercised power in influencing politicians to prioritise the disease [54]. Mexico and Chile have demonstrated the advantage of this inclusion in prioritising the disease and improving survival rates for common childhood cancers like acute lymphoblastic leukaemia [55–57]. India has also developed its own cooperative groups which include health care providers to address childhood cancer care, with efforts concentrating on clinical trials and training programs [58, 59]. This also encompasses engagement of grassroots organisations such as Cankids and Jiv Daya Foundation with state governments on identifying barriers to accessing care and potential solutions [60, 61]. Although this is a significant contribution to advance childhood cancer services in India, there needs to be greater concerted efforts in ensuring these services are prioritised on the national and state agendas and included within upcoming UHC policies.

Literature shows various strategies applicable for including childhood cancers within UHC, such as designing a “diagonal approach” of systems strengthening by synergizing disease specific approaches with horizontal (systemic) interventions [62], expanding financial coverage for treatment, building hospital and population-based registries [63], introducing community driven interventions such as parental education programs [64] and advancing research and advocacy [65]. Participants in our study had similar and specific examples of recommendations associated with strengthening primary care, developing community engagement on cancer care coordination through bottom-up efforts and advancing research and awareness. These recommendations are also in line with commentaries in 2011 that achieving UHC in India is limited without: public health funding and a bottom-up policy framework that includes addressing social determinants, community involvement and civil society engagement [66, 67]. Eight years later, similar suggestions are still echoed to improve access in India not just financially but holistically [68–70]. Some of the major impacts of Ayushman Bharat as stated by the government are the “timely treatments, improvement in health outcomes and patient satisfaction” [16]. Unfortunately, these impacts are not likely to greatly improve diagnosis, social supportive care or palliation of childhood cancers given the current focus of Ayushman Bharat is on financial risk protection for certain treatments and a plan for ‘health and wellness centres’ (which is yet to clearly outline the range of cancer services, if any). In addition, the National Multi-Sectoral Action Plan (2017–2022) outlines various programs for the prevention and control of NCDs [71]. Although this plan does not specify paediatric oncology, strengthening the health system to cater to common cancers might present an opportunity to indirectly impact the diagnosis and treatment for children with cancer.

Limitations of this study include: conducting the study in two cities and at the tertiary level of the health care system, as well as not including other key stakeholders in the paediatric haematology/oncology field who are part of advocacy strategies and policy reforms. We thus recommend undertaking qualitative studies in other cancer centres, with a wider range of stakeholders. This qualitative work could also be complemented with quantitative research on hospital readiness to treat children with cancer.

## Conclusion

In summary, we identified three key influences that shape the perception of health care providers about barriers to diagnosis and treatment for children with cancer: the type of hospital setting, the provider’s cadre, and individual factors. Policymakers may find this study useful in identifying the tangible and intangible barriers to accessing childhood cancer care, while recognising that perceptions of barriers may vary depending on stakeholder experiences, interests, and standpoints. Our findings reflect the importance of calls in global health to use qualitative research to increase understanding of barriers to accessing care and enhance UHC policies [27, 72–75]. It also contributes towards the World Health Organization’s recently launched Global Initiative for childhood cancers which aims to increase prioritisation and expand capacity to deliver services [76]. Insights from our findings may also be beneficial for understanding health system barriers to achieving UHC for other conditions, such as adult cancers which constitute an even larger disease burden in India.

## Supplementary Information

**Additional file 1: Table 1.** Topic guide used for the semi-structured interviews. Topic guide used for the semi-structured interviews.

**Additional file 2: Table 2.** Barrier themes, sub-themes and codes used in initial iterative analysis. A list of barrier themes, sub-themes and codes used in initial iterative analysis.

**Additional file 3: Table 3.** Names of Institutional Review Boards which approved the study. Names of Institutional Review Boards which approved the study.

## Data Availability

Full datasets analysed in this study are not publicly available due to risk of individual privacy being compromised and since the datasets are also being analysed as part of a doctoral thesis. Upon thesis examination, datasets may be available from the corresponding author upon reasonable request and with permission from the hospital Institutional Ethics Committees.

## Abbreviations

UHC: Universal Health Coverage
LMIC: Low- and middle-income country
NGO: Non-governmental organisation
