# Supplementary material for: Access to care for childhood cancers in India: perspectives of health care providers and the implications for universal health coverage
Source: BMC Public Health. 2020 Nov 3;20:1641. doi: 10.1186/s12889-020-09758-3 (PMC7607709; doi:10.1186/s12889-020-09758-3)
Supplement: Supplementary file 1 — Additional file 1: Table 1. Topic guide used for the semi-structured interviews. Topic guide used for the semi-structured interviews. [file 12889_2020_9758_MOESM1_ESM.docx]

## Additional File 1

Table 1**:** Topic guide used for the semi-structured interviews

| **1. Barriers to reaching care** |
| --- |
| a.     From the time parents first realize their child is unwell to the time they reach your centre what do you feel are the most significant challenges that families face in getting care for their child? |
| b.     For those patients who come to your center significantly later than would be ideal for initiation of optimal treatment, what do you feel could be done to change this situation? |
| c.     Are there preventative measures that have been put in place to help prevent occurrence of these issues? |
| d.     What additional services or resources do you feel are needed for patients to help them during their journey prior to arriving to your centre? |
| **2.     Barriers to Diagnosis** |
| a.     When patients are being diagnosed at this center do they face any significant challenges? If so, could you please tell me about what challenges you feel patients face most frequently? |
| **3.     Barriers to Initiating Treatment** |
| a.     When patients are initially beginning treatment in this center what are the greatest challenges that patients and their families experience? |
| b.     What resources or forms of support do patients have here that help them throughout the process of initiating and taking treatment? |
| **4.     Barriers to Continuing Treatment** |
| a.     For a patient who is taking treatment for a longer period of time what challenges do you feel that they and the family experience most frequently? |
| b.     What do you feel are the most frequent reasons why patients refuse or abandon the treatment? |
| **5.     Barriers to Palliative care & Survivorship** |
| a.     What do you think are the greatest challenges in providing palliative care to patients in your facility? |
| b.     What types of services or facilities are available to patients who have completed their treatment? |
| **6.     Resources** |
| a.     Please tell me about any support you receive from NGOs, trusts, or outside organizations which benefit pediatric oncology patients? |
| b.     What additional infrastructure and human resources do you wish were available to pediatric oncology patients in this center? |
| c.     What are the greatest challenges you face in providing care to pediatric cancer patients at this center in your role? |
| d.     What do you think are the resources that most significantly limit your ability to provide best possible care to pediatric oncology patients? |
| e.     Do you feel that additional training would be beneficial for the staff in providing better quality care to patients? |
| **7.     Other questions** |
| a.     What do you think about the current health system overall and how difficult/easy it is for patients with cancer and their families to navigate? |
| b.     Are you connected with other doctors treating pediatric oncology patients? Do you refer patients to other hospitals? |
| c.     From what type of physicians do you most commonly receive referred pediatric oncology patients? |
