# Supplementary material for: Access to care for childhood cancers in India: perspectives of health care providers and the implications for universal health coverage
Source: BMC Public Health. 2020 Nov 3;20:1641. doi: 10.1186/s12889-020-09758-3 (PMC7607709; doi:10.1186/s12889-020-09758-3)
Supplement: Supplementary file 2 — Additional file 2: Table 2. Barrier themes, sub-themes and codes used in initial iterative analysis. A list of barrier themes, sub-themes and codes used in initial iterative analysis. [file 12889_2020_9758_MOESM2_ESM.docx]

## Additional File 2

Table 2**:** Barrier themes, sub-themes and codes used in initial iterative analysis

| **Barrier themes and sub-themes** | **Codes** | **Sub-codes** |
| --- | --- | --- |
| Financial constraints   - Lack of personal money to start treatment - Lack of personal money to continue treatment - Lack of personal money for indirect costs such as food, travel, accommodation - Unable to obtain funding and insurance schemes - Funding/insurance schemes provided not adequate - Took a loan - Sold property - Loss of job, daily wages | FIN | FIN_I  FIN_HS |
| Social and cultural beliefs   - Lack of family support - Lack of community support - Fear of engaging with urban communities - Absence of caregiver - Gender bias - Preference for alternative treatment - Duration of treatment | SOC | SOC_I |
| Infrastructure   - Lack of beds for patients - Lack of basic hospital equipment for treatment - Lack of available drugs - Lack of appropriate diagnostic testing facilities - Long waiting times - Little or no psychological support for patients/caregivers - Difficulty in navigating health care system | INF | INF_HS |
| Health facility organization   - Medical staff organization - Methods of peer review - Methods of reimbursement - Hours of operation - Administrative delay - Cancer registry system | ORG | ORG_HS |
| Geographical issues   - Rural areas - Poor roads - Transportation difficulties - Difficult terrain - Distance - Lack of accommodation close to facility | GEO | GEO_I  GEO_HS |
| Service provision   - Lack of trained physicians - Lack of adequate physicians/nurses/social workers - Lack of treatment protocols/wrong treatment protocols - Protocols not followed - High patient load to service delivery facilities - Support programs for patients/caregivers | SER | SER_HS  SER_D |
| Awareness   - Lack of education - Lack of acceptance of condition - Lack of understanding of disease - Caregiver’s attitudes - Health care provider’s attitudes | AWA | AWA_I  AWA_HS  AWA_D |

Note: I = individual barriers, HS = health system barriers, D = disease barriers
