# Supplementary material for: Access to care for childhood cancers in India: perspectives of health care providers and the implications for universal health coverage
Source: BMC Public Health. 2020 Nov 3;20:1641. doi: 10.1186/s12889-020-09758-3 (PMC7607709; doi:10.1186/s12889-020-09758-3)
Supplement: Supplementary file 3 — Additional file 3: Table 3. Names of Institutional Review Boards which approved the study. Names of Institutional Review Boards which approved the study. [file 12889_2020_9758_MOESM3_ESM.docx]

Table 3: Names of Institutional Review Boards and ethics reference numbers for each hospital which approved the study (in no particular order).

a) Institutional Ethics Committee of Max Super Speciality Hospital
(Ref no: RS/MSSH/SKT-2/ONCO/IEC/17-37)

b) Institutional Ethics Committee of Indraprastha Apollo Hospital
(Ref no: IAH/091/06-17)

c) Institutional Ethics Committee of Institute Rotary Cancer Hospital, All India Institute of Medical Sciences
(Ref no: IEC-387/07.07.2017)

d) Institutional Ethics Committee of Basavatarakam Indo American Cancer Hospital
(Ref no: IEC/2017/153)

e) Institutional Ethics Committee of MNJ Cancer Hospital
(Ref no: ECR/227/Inst/AP/2013/RR-16)

f) Institutional Ethics Committee of Rainbow Children’s Hospital
(Ref no: RCHBH/044/08-2017)

g) Institutional Ethics Committee of All India Institute of Medical Sciences
(Ref no: IEC-615/03.11.2017, RP-17/2017)
